# Risk factors for metabolic bone disease among preterm infants less than 32 weeks gestation with Bronchopulmonary dysplasia

**DOI:** 10.1186/s12887-021-02705-0

**Published:** 2021-05-17

**Authors:** Wenwen Chen, Zhenhai Zhang, Shuzhen Dai, Liping Xu

**Affiliations:** Zhangzhou Hospital Affiliated to Fujian Medical University, Shengli W Rd, Xiangcheng District, Zhangzhou, Fujian China

**Keywords:** Metabolic bone disease, Bronchopulmonary dysplasia, Preterm, Risk factors

## Abstract

**Background:**

Bronchopulmonary dysplasia (BPD) infants present an increased incidence of metabolic bone disease (MBD), but it is unknown which factors contribute to this. The aim of this study was to determine the risk factors for developing MBD in BPD infants.

**Methods:**

A retrospective review of the medical records of BPD infants admitted to the Neonatal intensive care unit at Zhangzhou Hospital between Jun 2016 and May 2020 was performed. BPD infants with MBD were identified, two contemporaneous without MBD matched by gestational age and gender were randomly selected as controls for each case of MBD. The association between putative risk factors and MBD was estimated with ORs and 95% CIs. A *P*-value threshold ≤0.2 was used in univariate analysis for inclusion into a multivariate (adjusted) model with a *P*-value of < 0.05 as statistically significant.

**Results:**

A total of 156 BPD infants were enrolled with 52 cases of MBD and 104 controls. Fetal growth restriction (OR 6.00, 95% CI, 1.81–19.84), extremely low birth weight (OR 3.10, 95% CI, 1.07–8.94), feeding volume < 80 mL/kg/d at the end of the 4**th** week after birth (OR 14.98, 95% CI, 4.04–55.58), cholestasis (OR 4.44, 95% CI, 1.59–12.40), late onset sepsis (OR 3.95, 95% CI, 1.12–13.98) and prolonged (> 2 weeks) diuretics application (OR 5.45, 95% CI, 1.25–23.84) were found to be statistically significant risk factors for MBD in BPD infants.

**Conclusion:**

In BPD infants of homogeneous gestational age, fetal growth restriction, extremely low birth weight, feeding volume < 80 mL/kg/d at the end of the 4th week after birth, cholestasis and late onset sepsis are significant risk factors for MBD. These findings provide potential predictive factors for MBD in BPD infants and warrant prospective validation.

## Background

Metabolic bone disease (MBD), characterized by low bone mass and demineralization of bone tissue, affects approximately 16–40% of very low birth weight (VLBW, birth weight < 1500 g) and extremely low birth weight (ELBW, birth weight < 1000 g) infants [1]. The exact incidence of MBD varied in different centers from a lack of consensus on the definition of MBD. MBD poses a consequent increase in bone fragility, contributing to long-term reduced linear growth. As MBD advances, rachitic changes and/or fractures are frequently observed. The understanding of MBD has grown in recent years, along with other preterm comorbidities. It has been reported that infants with bronchopulmonary dysplasia (BPD) have a higher incidence of MBD [2]. Among infants with birth weight < 1500 g suffered from severe BPD, about one third developed severe MBD [3].

Several factors contributed to MBD had been identified. Costa R et al. found that the majority of MBD infants were male and presented lower gestational age, lower birth weight, and prolonged duration of parenteral nutrition [4]. Chen W et al. found that lower gestational age (< 30 weeks), vitamin D supplementation at > 14 days of age, and achievement of total enteral nutrition (TEN) beyond 28 days of age were independent risk factors for MBD in infants < 34 weeks gestational age [5]. Montaner Ramón A et al. found that only restricted fetal growth was independently associated with the development of severe MBD (OR 9.65, 95% CI 3.48–26.76) in infants < 32 weeks gestational age and/or weight < 1500 g [6]. Ukarapong S et al. found that only cholestasis remained significantly risk factors (OR 9.6, 95% CI 2.1–45.3) in infants < 30 weeks gestational age and/or weight < 1, 000 g [7]. Although BPD infants presented an increased risk for developing MBD, previous studies did not evaluate the risk factors that would be associated with MBD in BPD infants. On the other hand, some risk factors for MBD might become not obvious in infants with BPD since demographic and complications could be more homogeneous.

The present study was designed with the objective of knowing the risk factors for MBD in infants with BPD. These risk factors will help to identify high-risk individuals and propose recommendations for the prevention of MBD.

## Methods

### Study population and setting

The study was conducted in Zhangzhou Hospital with retrospective review of the electronic medical record-derived data of BPD infants who admitted to the Neonatal intensive care unit (NICU) between Jun 2016 and May 2020. The protocol was approved by the Institutional Ethical Committee of the hospital, with waiver of informed consent. BPD infants with MBD were identified as case group. Inclusion criteria consisted of the following: (1) gestational age < 32 weeks, (2) survival up to 36 weeks post-menstrual age (PMA), (3) diagnosis of BPD according to National Institutes of Health that defined as requirement of oxygen support (> 21%) for at least 28 days and a subsequent assessment at 36 weeks PMA or discharge, whichever comes first. At the time of assessment, infants with no oxygen requirement were classified as having mild BPD, infants requiring < 30% oxygen were classified as having moderate BPD and infants with a need for positive pressure ventilation/continuous positive pressure and/or oxygen requirement ≥30% were classified as having severe BPD [8], and (4) diagnosis of MBD that defined as peak serum alkaline phosphatase (ALP) higher than 900 U/L and serum phosphorus ower than 1.8 mmol/L, which yielded a sensitivity of 100% at a specificity of 70%, with or without radiographic changes of long bones [9]. Exclusion criteria included: (1) gestational age ≥ 32 weeks, (2) presence of significant congenital anomalies, including malformations of the digestive tract, (3) death before 36 weeks PMA. In our unit, mortality of infants with gestational age < 32 weeks after 36 weeks PMA was 3.2%. The incidence of MBD among BPD infants was 11.8%. For each MBD case, two contemporaneous with BPD but without MBD matched at equivalent gestational age and gender (male: female = 1:1) were randomly selected as controls. The retrospective nature of the study predetermines the actual sample size.

Clinical data on demographics and putative risk factors for MBD were collected, including birth weight, gender, use of antenatal steroids, histologic chorioamnionitis (HCA), fetal growth restriction (FGR), maternal hypertensive disorders without FGR, prolonged rupture of membranes (PROM), type of feeding, initiation of oral vitamin D supplementation, feeding volume at the end of the 4th week after birth, necrotizing enterocolitis (NEC), patent ductus arteriosus (PDA), cholestasis, late onset sepsis (LOS), postnatal dexamethasone, diuretics application, fluid restriction, mechanical ventilation, thyroid function tests and platelet (PLT) count.

FGR was defined as antenatal diagnosis by any 2 of the following: estimated fetal weight below the 10th percentile according to the care provider reference curve, abnormal fetal Doppler findings, growth arrest, or maternal hypertensive disorders [10]. PROM was defined as rupture of the membranes occurring earlier than 24 h before delivery [10]. HCA was defined as the infiltration of neutrophils in any of the following structures: placental disc, the chorioamniotic membranes, and the umbilical cord [10]. Cholestasis was defined as direct bilirubin > 2 mg/dL [11]. Maternal hypertensive disorder was defined as systolic blood pressure ≥ 140 mmHg and (or) diastolic blood pressure ≥ 90 mmHg [12]. NEC was diagnosed according to revised Bell staging criteria. PDA was diagnosed with echocardiography confirmation beyond 3 days after birth. Hemodynamically significant PDA (hsPDA) was defined as a duct size > 1.5 mm and a left atrium-to-aortic root (LA:Ao) > 1.5 and/or left-to-right shunting of blood, or end-diastolic reversal of the blood flow in the aorta [13]. Thyroid-stimulating hormone > 10 mU/L was used as the cut-off level for hypothyroidism [14] and PLT < 100 × 10^9/L was considered to be thrombocytopenia [15]. LOS was diagnosed with clinical suspicion and evaluated by microbial culture or two non-specific blood indicators occurring after 72 h of birth [16].

In our NICU, we started parenteral nutrition within the first hours after birth and introduced enteral feeding with breast milk as soon as the infant reached a tolerated respiratory rate without hemodynamic instability. The feeding amount increased gradually with a rate of 20–30 mL/kg/d without signs of feeding intolerance. Oral vitamin D (800–1000 IU/d) supplementation was started when the feeding amount reaches 50 mL/kg/d, and human milk fortification was added when the feeding amount of breast milk reaches 80 mL/kg/d. Premature formula was also used if the amount of breast milk was insufficient. During hospitalization period, starting from the 2nd day after birth, while maintaining parenteral nutrition, serum calcium, phosphorus and ALP were measured weekly, and changed to twice a week after the establishment of TEN.

Regarding postnatal diuretics application, loop diuretics (furosemide) was administrated occasionally when intake volume exceeded output remarkably. Fluid restriction (total fluid volume < 140 mL/kg beyond 2 weeks after birth) was introduced when there was pulmonary edema on chest radiographs. Furosemide (or hydrochlorothiazide) and spironolactone were applied when pulmonary edema was still obvious after fluid restriction. Diuretics at any type (including single dose and multiple doses) and prolonged duration (> 2 weeks) were analyzed differently.

### Statistical analyses

Data were analyzed using SPSS version 26 and GraphPad Prism version 8 software. Descriptive data were presented as means±SD or medians (IQR) or percentages. Dichotomous or categorical variables were compared between cases and controls with the use of the χ^2^ analysis or Fisher exact test or z-test. Continuous variables were compared between groups via the two-sample t-test or Mann-Whitney *U* test. A risk model was derived via logistic regression, with 95% CIs calculated for ORs using the forced entry method. A *P*-value of ≤0.2 was used in univariate analysis for inclusion of putative risk factors into the multivariate (adjusted) model. A *P*-value of < 0.05 was considered statistically significant.

## Results

**Fig. 1:**
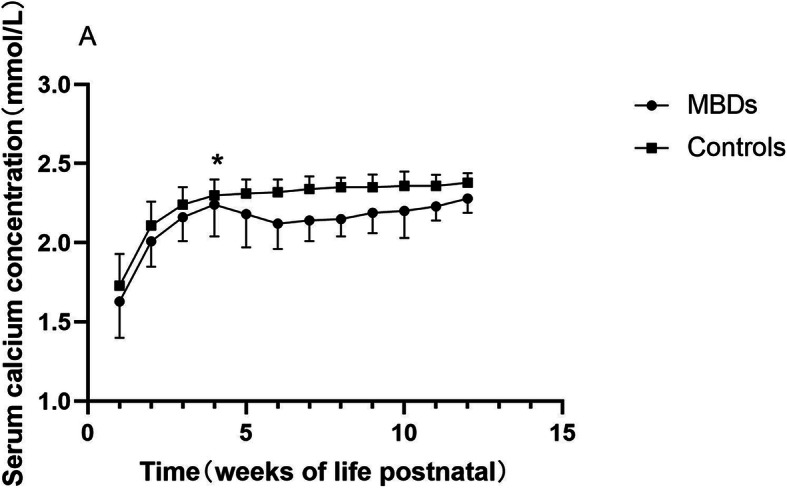
Serum calcium concentration by weeks of life

**Fig. 2:**
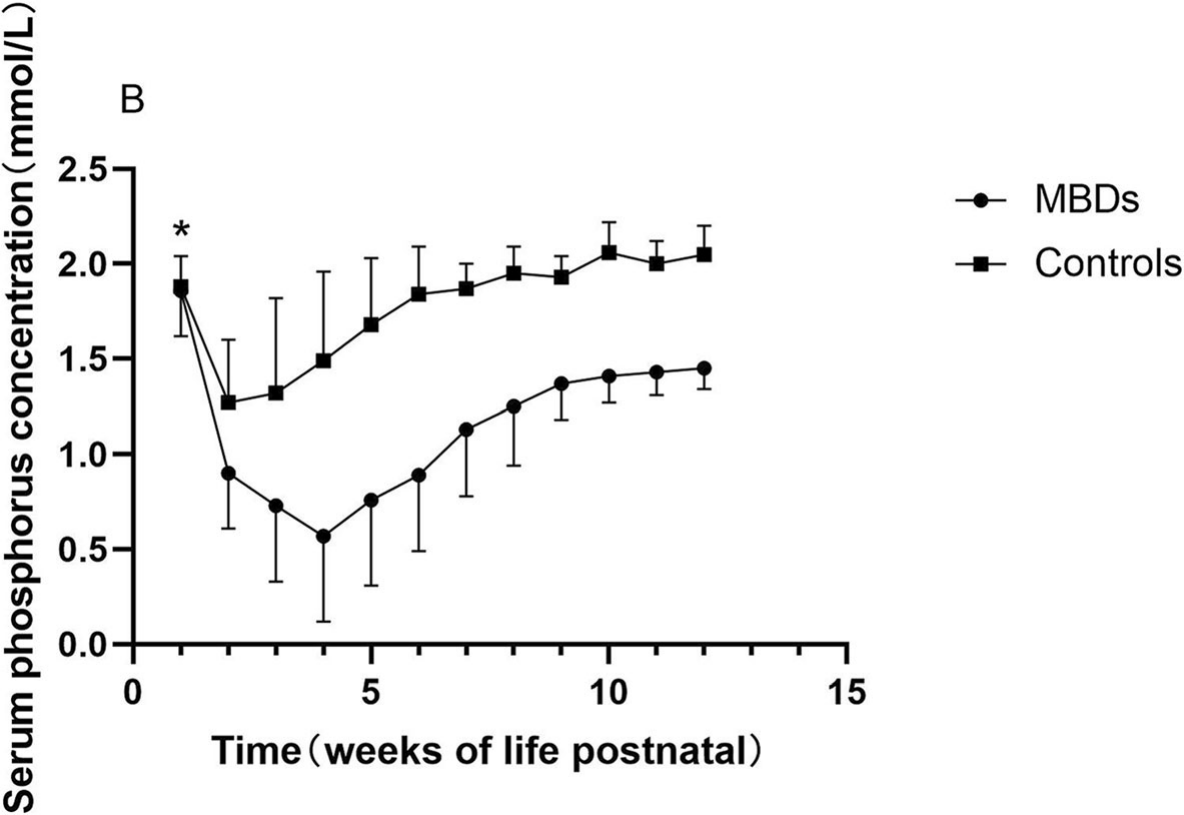
Serum phosphorus concentration by weeks of life

A total of 156 infants with BPD were enrolled in the study, with 52 cases of MBD and 104 controls. Radiographic changes including loss of metaphyseal sclerotic line, thinning of diaphyseal cortical, osteopenia, splaying, fraying, cupping or coarse trabecular pattern of metaphyses were found in 30.8% (16/52) of the MBD infants, while none of the controls showed apparente radiographic abnormalities. Gestational age was similar among the two groups (MBD 28.5 ± 1.6 w vs controls 28.5 ± 1.6 w, *P* = 0.915). MBD infants had a lower birth weight (MBD 1051 ± 186 g vs controls 1207 ± 221 g, *P* < 0.001) compared with controls. There was no significant difference between the two groups in the distribution of BPD severity (mild BPD 59.6% vs. 70.2%; moderate BPD 23.1% vs 21.2%; severe BPD 17.3%vs 8.7% in MBD infants and controls respectively; *P* = 0.236). Baseline serum calcium was lower in MBD infants, while baseline serum phosphorus did not show significantly difference (shown in Fig. 1 and 2. Figure note: Figure 1 Serum calcium concentration by weeks of life. Figure 2 Serum phosphorus concentration by weeks of life. Numbers on time axis refers to weeks postnatal. Data within first week postnatal were acquired on the 2nd day after birth. * *P* > 0.05.). Serum calcium elevated, while phosphorus had an apparente decline preceding the ascension after birth. Compared with controls, MBD infants had a delay in phosphorus ascension and durative lower phosphorus concentration from the 2nd week after birth. Interestingly, serum calcium got very similar in both groups at the 4th week after birth, and then MBD infants had a sharp down, while controls maintained at stable level. Other demographic and clinical characteristics among cases and controls presented as categorical variables were shown in Table 1.

**Table 1:** Demographic and clinical characteristics among cases with MBD and controls

	Cases		Controls	
Antenatal factors	n	%	n	%
	52		104	
Male	28	53.8	52	50.0
Steroids	37	71.2	79	76.0
HCA	29	55.8	50	48.1
FGR	16	30.8	7	6.7
Maternal hypertensive disorders without FGR	5	9.6	11	10.6
PROM	11	21.2	27	26.0
Postnatal factors				
ELBW	16	30.8	17	16.3
Exclusive breast milk fed	39	75.0	71	68.3
Initiation of oral vitamin D supplementation ≥2 weeks after birth	20	38.5	26	25.0
Feeding volume<80 mL/kg/day at the end of the 4th week after birth	23	44.2	6	5.8
NEC	5	9.6	4	3.8
PDA	25	48.1	47	45.2
hsPDA	21	40.4	35	33.7
Cholestasis	22	42.3	18	17.3
LOS	12	23.1	8	7.7
Moderate-severe BPD	31	40.4	21	29.8
Mechanical ventilation	28	53.8	57	54.8
Mechanical ventilation at 36 PMA	4	7.7	2	1.9
Dexamethasone	11	21.2	14	13.5
Diuretics	16	30.8	29	27.9
Prolonged diuretics (>2 weeks)	10	19.2	4	3.8
Fluid restriction (<140 mL/kg beyond 2 weeks after birth)	13	25.0	19	18.3
Hypothyroidism	15	28.8	24	23.1
Thrombocytopenia	9	17.3	10	9.6

MBD, metabolic bone disease; HCA, histologic chorioamnionitis; FGR, fetal growth restriction; PROM, prolonged rupture of membranes; ELBW, extremely low birth weight; NEC, necrotizing enterocolitis; PDA, patent ductus arteriosus; hsPDA, hemodynamically significant PDA; LOS, late onset sepsis; BPD, bronchopulmonary dysplasia; PMA, post-menstrual age

Univariate logistic regression analyses (Table 2) revealed that, among the putative risk factors evaluated, FGR (OR 6.16, 95% CI, 2.34–16.20), ELBW (OR 2.49, 95% CI, 1.14–5.41), feeding volume < 80 mL/kg/d at the end of the 4th week after birth (OR 12.95, 95% CI, 4.82–34.84), cholestasis (OR 3.50, 95% CI, 1.66–7.41), LOS (OR 3.60, 95% CI, 1.37–9.47) and prolonged (> 2 weeks) diuretics application (OR 5.95, 95% CI, 1.77–20.05) were all associated with the development of MBD in BPD infants. After adjustment in multiple logistic regression, each remained as statistically significant, independent risk factors (FGR [OR 6.00, 95% CI, 1.81–19.84], ELBW [OR 3.10, 95% CI, 1.07–8.94]), feeding volume < 80 mL/kg/d at the end of the 4th week after birth [OR 14.98, 95% CI, 4.04–55.58], cholestasis [OR 4.44, 95% CI, 1.59–12.40], LOS [OR 3.95, 95% CI, 1.12–13.98], prolonged diuretics application[OR 5.45, 95% CI, 1.25–23.84]).

**Table 2:** Unadjusted OR and aOR for putative risk factors for development of MBD in BPD infants from univariate and multiple logistic regression

	Unadjusted				Adjusted			
Putative risk factors		95%	95%			95%	95%	
	OR	LCL	UCL	P	OR	LCL	UCL	P
Antenatal factors								
Steroids	0.78	0.37	1.65	0.517				
HCA	1.36	0.70	2.66	0.366				
FGR	6.16	2.34	16.20	<0.001	6.00	1.81	19.84	0.003
Maternal hypertensive disorders without FGR	0.90	0.30	2.74	0.852				
PROM	0.77	0.35	1.70	0.765				
Postnatal factors								
ELBW	2.49	1.14	5.41	0.022	3.10	1.07	8.94	0.037
Exclusive breast milk fed	0.72	0.34	1.52	0.386				
Initiation of oral vitamin D supplementation ≥2 weeks after birth	1.98	0.96	4.05	0.063	1.24	0.43	3.53	0.691
Feeding volume <80 mL/kg/day at the end of the 4th week after birth	12.95	4.82	34.84	<0.001	14.98	4.04	55.58	<0.001
NEC	2.66	0.68	10.36	0.159	0.45	0.06	3.61	0.449
PDA	1.12	0.58	2.19	0.733				
hsPDA	1.34	0.67	2.66	0.409				
Cholestasis	3.50	1.66	7.41	0.001	4.44	1.59	12.40	0.004
LOS	3.60	1.37	9.47	0.009	3.95	1.12	13.98	0.033
Dexamethasone	1.73	0.72	4.12	0.220				
Diuretics	1.15	0.56	2.38	0.708				
Prolonged diuretics (>2 weeks)	5.95	1.77	20.05	0.004	5.45	1.25	23.84	0.024
Fluid restriction (<140 mL/kg beyond 2 weeks after birth)	1.49	0.67	3.32	0.328				
Mechanical ventilation	0.96	0.49	1.88	0.909				
Hypothyroidism	1.35	0.64	2.87	0.434				
Thrombocytopenia	1.97	0.75	5.20	0.172	0.75	0.15	3.84	0.729

MBD, metabolic bone disease; BPD, bronchopulmonary dysplasia; HCA, histologic chorioamnionitis; FGR, fetal growth restriction; PROM, prolonged rupture of membranes; ELBW, extremely low birth weight; NEC, necrotizing enterocolitis; PDA, patent ductus arteriosus; hsPDA, hemodynamically significant PDA; LOS, late onset sepsis

The ratio of different feeding types was not significantly different between the two groups (exclusive breast milk fed 75.0% vs. 68.3%; partial formula fed 13.5% vs 21.2%; exclusive formula fed 11.5%vs 10.6% in MBD infants and controls respectively; *P* = 0.508). At the end of the 4th week after birth, MBD infants received a less feeding volume (MBD 107.3 ± 49.3 ml vs. controls 142.2 ± 27.8 ml, *P* < 0.001) compared with controls. MBD infants had a delay in time to start fortification [MBD 17(9, 30) d vs. controls 9(8, 14) d, *P* < 0.001] compared with controls.

No significant difference was found in the need for invasive mechanical ventilation at 36 weeks PMA [MBD 7.7% vs. controls 1.9%, *P* = 0.185], or the length of invasive mechanical ventilation [MBD 2(0,14) d vs. controls 1(0,5) d, *P* = 0.238] between the two groups.

## Discussion

The growing advances in the intensive care of preterm infants have led to a decrease in mortality, but caused more frequent morbidity such as MBD and BPD, etc. Previous studies have proved that MBD is inversely associated with gestational age [4, 5], hence we matched the gestational age equal to the cases when selecting the controls in order to evaluate other putative factors in homogeneous groups. This study provides novel data on risk factors for MBD in BPD infants. FGR, ELBW (birth weight < 1000 g), feeding volume < 80 mL/kg/d at the end of the 4th week after birth, cholestasis and LOS each served as statistically significant, independent risk factors, on the basis of their associated ORs from multiple logistic regression.

The association between FGR and MBD in BPD infants was confirmed in our study which was in accordance with other data published [8]. This early life factor seemed to ease MBD development in a programmed process, which could be used as an early predictive indicator for screening of MBD [17]. FGR commonly coexists with preeclampsia at gestation, resulting in placental abnormalities that deteriorates placental transfer of calcium, magnesium and phosphorus [18]. Furthermore, our study showed that maternal hypertensive disorder without FGR was not associated with MBD, indicating that the disrupted placental function might be the key pathogenesis. In addition to FGR, ELBW was also an independent risk factor for MBD in BPD infants of equal gestational age in our study samples, revealing an inverse relation between birth weight and MBD, which was found even in ELBWs exclusively by Viswanathan S et al. [19].

Changes in mineral supply, hormonal environment and mechanical stress may cause disrupted bone metabolism after birth. In the early stage, serum calcium can maintain within the optimum range, while phosphorus declined at 1–2 weeks after birth. This could be physiologically and self-limited since infants without MBD took on the same biochemical changes. However, MBD infants presented a longer down period, and lower levels of serum phosphorus and calcium ultimately. Prolonged hypophosphatemia might be more meaningful characteristics and indicator for MBD.

Feeding problems are almost inevitable in the very preterm infants. Generally, it is not difficult to introduce enteral feeding but hard to reach TEN because uncomfortable abdominal distention, gastric retention or signs of NEC usually disrupt the feeding schedule. Prolonged parenteral nutrition (PN) was demonstrated to be associated with MBD by several research elsewhere [20]. Similarly, our study revealed that an enteral feeding volume < 80 mL/kg/d at the end of the 4th week after birth remarkably increased the risk for MBD in BPD infants. This may be explained by the following reasons. First, the small feeding volume of breast milk did not allow the introduce of fortifier in our NICU. Even at the usual feeding volume, breast milk did not provide sufficient protein and mineral content to guarantee enough calcium and phosphate intake for preterm infants. Second, the insufficient feeding volume still required PN supplement which posed the possibility of aluminum contamination and the risk of mineral precipitation in the solution due to the small volumes [1]. It has been demonstrated that newborns fed exclusively with breast milk showed lower levels of phosphate [21] and fortified breast milk feeding significantly increase bone mineral density values via linear regression analysis [22]. Therefore, an earlier begin of fortification of breast milk might be taken into consideration in breast-fed preterm infants with high risk for BPD, as appropriate. Some studies showed that vitamin D supplementation at > 14 days of age was also associated with MBD, but the results of our study were inconsistent with it. A possible explanation is that it might be the serum 25(OH)D3 that is directly associated with MBD [6]. A usual intake dose of 800–1000 IU/d for preterm infants with BPD did not achieve the protective level, which is probable due to fat-soluble vitamin deficiency caused by the coexisting cholestasis [23]. Simultaneously, cholestasis was also a risk factor for MBD in BPD infants confirmed by our study and other research [20]. In other words, these infants may be at a greater demand for vitamin D supplementation.

LOS remains one of the most common causes of morbidity and mortality in preterm infants [24]. Jensen EA et al. found that blood culture confirmed sepsis was associated with increased odds of severe MBD in infants with severe BPD [3]. Our study reenforced the association between LOS and MBD in BPD infants. It has been acknowledged that important interactions occur between immune and skeletal systems [25]. Lipopolysaccharide exposures could result in bone loss [26], which might be due to the activation of B and T cells that potentially regulate bone resorption [25]. Approaches such as strict hygiene protocol, minimization of invasive interventions and probiotic supplementation in exclusively breast-fed preterm [27] should be employed in prevention of LOS, as promising measures to reduce MBD.

Diuretics are widely used in NICU with the expectations of improvement in pulmonary mechanics and avoidance of excessive weight gain [28]. However, there are not enough evidence supporting their long-term benefits considering improvement in mortality, duration of mechanical ventilation or oxygen dependence [29]. Furthermore, potential effects of diuretics on bone have been recognized. A single daily dose of loop diuretics (LDs) could increase renal calcium excretion and alter the diurnal rhythm of plasma parathyroid hormone (PTH) levels, and consecutive doses may ultimately result in elevation of circulating PTH and hypophosphatemia [30]. Our study provided an important look at that the duration of LDs therapy in BPD infants was associated with MBD. Orth LE el al also demonstrated that infants exposed to increased cumulative LDs exposure had a higher incidence of MBD and suggested a late diuretic administration initiated after 2 weeks of life [31]. However, serum phosphorus began to decline from 1 to 2 weeks after life, and the declining could last for several weeks. Diuretics usage in this stage might cause adverse effect on mineral metabolism additively. These factors should be considered when making decision to use diuretics in BPD infants.

The strengths of this study are as follows. First, we enrolled only infants with BPD and matched gestational age to ensure the comparison in homogeneous groups. Second, the monocentric study guaranteed that the enrollments were managed strictly under the same perinatal practices including diagnosis and treatments. But there are some limitations. Since MBD is a multifactorial disease, the small sample size may not cover all important risk factors. However, we determine some risk factors that could be interpreted. The results in our study revealed that MBD in BPD infants shared some risk factors the same as preterm infants in general.

## Conclusion

In BPD infants of homogeneous gestational age, FGR, ELBW, feeding volume < 80 mL/kg/d at the end of the 4th week after birth, cholestasis, LOS and prolonged (> 2 weeks) diuretics application are significant risk factors for MBD. Given the observational nature of this study, further longitudinal/prospective studies are required to validate these findings.

## Data Availability

The datasets used and/or analyzed during the current study are available from the corresponding author on reasonable request.

## Abbreviations

MBD: Metabolic bone disease
BPD: Bronchopulmonary dysplasia
HCA: Histologic chorioamnionitis
FGR: Fetal growth restriction
ELBW: Extremely low birth weight
VLBW: Very low birth weight
PROM: Prolonged rupture of membranes
PDA: Patent ductus arteriosus
hsPDA: Hemodynamically significant PDA
NEC: Necrotizing enterocolitis
LOS: Late onset sepsis
ALP: Alkaline phosphatase
PTH: Parathyroid hormone
PLT: Platelet
LDs: Loop diuretics
PMA: Post-menstrual age
TEN: Total enteral nutrition
PN: Parenteral nutrition
NICU: Neonatal intensive care unit
OR: Odds ratio
aOR: Adjusted odds ratio
CI: Confidence interval
